# A review of significant events analysed in general practice: implications for the quality and safety of patient care

**DOI:** 10.1186/1471-2296-10-61

**Published:** 2009-09-01

**Authors:** John McKay, Nick Bradley, Murray Lough, Paul Bowie

**Affiliations:** 1 Division of Community Based Sciences, University of Glasgow, Glasgow, UK; 2 NHS Education for Scotland, Glasgow, UK

## Abstract

**Background:**

Significant event analysis (SEA) is promoted as a team-based approach to enhancing patient safety through reflective learning. Evidence of SEA participation is required for appraisal and contractual purposes in UK general practice. A voluntary educational model in the west of Scotland enables general practitioners (GPs) and doctors-in-training to submit SEA reports for feedback from trained peers. We reviewed reports to identify the range of safety issues analysed, learning needs raised and actions taken by GP teams.

**Method:**

Content analysis of SEA reports submitted in an 18 month period between 2005 and 2007.

**Results:**

191 SEA reports were reviewed. 48 described patient harm (25.1%). A further 109 reports (57.1%) outlined circumstances that had the potential to cause patient harm. Individual 'error' was cited as the most common reason for event occurrence (32.5%). Learning opportunities were identified in 182 reports (95.3%) but were often non-specific professional issues not shared with the wider practice team. 154 SEA reports (80.1%) described actions taken to improve practice systems or professional behaviour. However, non-medical staff were less likely to be involved in the changes resulting from event analyses describing patient harm (p < 0.05)

**Conclusion:**

The study provides some evidence of the potential of SEA to improve healthcare quality and safety. If applied rigorously, GP teams and doctors in training can use the technique to investigate and learn from a wide variety of quality issues including those resulting in patient harm. This leads to reported change but it is unclear if such improvement is sustained.

## Background

Patient safety dominates the agenda in most modern health care systems including the National Health Service (NHS) in the United Kingdom (UK) [1,2]. However, evidence of the main threats to patient safety and their potential solutions is limited. Although incidences of error and rates of patient harm have been quantified to some extent in secondary care [3-7] similar estimates in primary care [8-18] have been criticised as lacking in consistency and theoretical construct [19].

Despite many safety concerns being generic across healthcare sectors, primary care is recognised as involving specific challenges. It is characterised by self-limiting conditions where safety-critical incidents occur relatively infrequently and clinical management decision-making often includes a level of uncertainty as a result of often undifferentiated symptoms across a broad range of health and illness issues [20,21]. The differing physical, psychological, social and personal choices unique to each patient mean that there is often justifiable variation in practice [22,23]. Similarly, the unique business position of UK general practices (independent contractor status) compared with the acute sector (largely directly managed NHS organisations) is an unknown quantity in terms of influence and impact on patient safety concerns. Quality improvement methods when applied in the general practice context may need to allow for these variations, complexities and associated organisational and cultural factors.

One potential improvement method that is largely, although not exclusively, confined to UK primary care is significant event analysis (SEA) [24] which is promoted as a team-based approach to enhancing safety, managing risk and facilitating the reporting of safety incidents [25-27]. Expectations for SEA are high. Individual general practitioners (GPs) and their health care teams must provide documentary evidence of SEA participation as part of professional, contractual and clinical governance obligations [28,29]. The National Patient Safety Agency (NPSA) also recommends that primary care teams should identify and analyse those significant events which have resulted in "minor" or "moderate" harm to patients, or had the potential to do so [27].

However, two interrelated issues arguably hinder the progress of the safety agenda in general practice. Firstly, there is a lack of evidence for the effectiveness of SEA in terms of its value in facilitating learning and change which leads to credible improvements in health care quality [25]. Secondly, fully engaging the primary care team in the reporting of patient safety incidents as part of formal reporting and learning mechanisms has proved to be extremely challenging [30].

Gaining some insight into how SEA may contribute to our understanding of important quality and safety issues is clearly desirable to help close the evidential gap. In the west of Scotland region, a voluntary educational system offers GPs the opportunity to submit SEA reports for peer feedback from trained colleagues as part of continuing professional developments [31]. A substantial bank of reports has been submitted and retained in the past decade, which offers important potential for research and learning.

Against this background, the aim of this study was to review the contents of SEA reports submitted by GPs and in doing so to identify the range of quality and safety issues analysed, the types of learning needs raised and the purported actions implemented by health care teams. The findings could then be used to inform on aspects of the debate on the effectiveness of SEA [25].

## Methods

### Study design, sample, timescale and ethical approval

The study involved a content analysis of SEA reports voluntarily submitted by GPs between July 2005 and February 2007 for external peer feedback as part of an educational model developed and managed by NHS Education for Scotland (NES) - a special health authority with responsibility for the training and education of the healthcare workforce [31]. In this model a significant event is defined as *"...any event thought by any member of the practice team to be significant in the care of patients or the conduct of the practice*" [24]. This broad definition was to allow for any issue that impacts either directly or indirectly on the quality of patient care to be addressed.

The study was approved by NHS Greater Glasgow Primary Care Division Research Ethics Committee (REC Ref No. 04/S0701/71).

### Peer review of SEA reports

All reports were submitted by two GP groups: GP principals (qualified family doctors) as part of their continuing professional development and GP registrars (family doctors in training) as part of a regional training requirement.

SEA were submitted in a standard report format (Table 1) and sent to two trained GPs for independent review using a validated feedback instrument [32]. Good practice in SEA recommends that it involves all necessary members of the healthcare team and is conducted in a fair and non-threatening environment. As such, the author of the report is encouraged to describe the consensus view of the team's analysis where appropriate.

**Table 1:** Summary of standard SEA framework and report format recommended in NHS Scotland

**1. What Happened?**
• Collate and record as much factual information as possible about the event including, for example, what happened, when and where, what was the outcome and who was involved.
• Record the thoughts and opinions of those involved, including patients and relatives if appropriate, and attempt to form an accurate impression of what happened
**2. Why did it happen?**
• Ensure the main reasons why the event occurred are fully established and recorded, e.g. was it a failure in a system or a failure to adhere to protocol?
• Establish the underlying or contributory reasons as to why the event occurred, e.g. why was there a failure in a system or adherence to a protocol.

**3. What has been learned?**
• Agree and record the main learning issues for the health care team or individual team members.
• Ensure that insight into the event has been established by the team or the individuals concerned

**4. What has been changed?**
• Agree and implement appropriate action in order to minimize the chance of recurrence, where change is considered to be relevant.
• Monitor the implementation of any change introduced

As part of an overall global judgement on the quality of the analysis report, reviewers were asked to make a determination using the feedback instrument as to whether the SEA report was satisfactory or unsatisfactory. Where there was disagreement between the two reviewers a third reviewer - the peer review co-ordinator - would make a final decision. Those considered 'unsatisfactory' were excluded from the study because they were highly likely to be deficient analyses. We know from previous research involving these 'unsatisfactory' reports that insights into why the event happened may be lacking, learning needs may not have been identified or appropriate action was not taken to reduce the risk of recurrence [33]. All GPs consented to their anonymised reports being used in this study.

### Content analysis of SEA reports

#### Coding of events, reasons for occurrence, learning needs and actions taken

Each SEA report was analysed for content independently by two researchers (NB & JM). The authors developed a preliminary coding framework by merging and adapting the main categories and subcategories of errors, [12,15] adverse events [10] and potentially harmful events [9] previously reported in published research from primary care. The codings were further developed and refined as the study progressed. The authors were unaware of similar frameworks for classifying learning needs and actions taken. These were developed on an iterative basis as each SEA report was reviewed. Reports often described multiple events, reasons for occurrence, learning needs and actions taken. A pragmatic decision was taken not to assign a single code for each of these factors because of the inherent difficulty in reaching agreement because events could be highly complex, information could be missing and improvements were often multi-factorial.

### Data validation

Joint meetings between the researchers took place after a set of five SEA reports had been reviewed. Where there was disagreement between researchers a consensus was reached on the codes assigned. To enhance validity, a third researcher (PB) independently analysed one-in-five reports and the associated coding before meeting with the other two researchers to triangulate final agreement on the data collected.

### Data collection and analysis

The following data were collected using a pre-designed proforma: type of significant event; reasons cited for event occurrence; involvement of external agencies/individuals; level of patient harm (NPSA grading system: death, severe, moderate, low and none); type of learning issues identified; and type of actions taken. Data were entered into a Microsoft Excel spreadsheet. Descriptive statistics and differences in proportions between GP group data were calculated along with 95% confidence intervals using Minitab version 13.

## Results

### SEA reports reviewed

286 SEA reports were submitted during the study timescale. Of these 95 (33%) were excluded because they were judged 'unsatisfactory' by peer review. A total of 191 SEA reports were therefore subject to review of which a 99 were submitted by 74 GP principals (51.8%) {Range one to four reports per GP principal} and 92 by 90 GP registrars (48.1%) {Range one to two reports per GP registrar}.

### Types of significant event

A classification summary of the most frequently occurring significant event codes with examples is outlined in Table 2. Events involving disease diagnoses and/or disease management (46, 24.1%) along with prescribing issues (46, 24.1%) were the most common subjects for analysis. 'Disease diagnosis' categorisation involved issues such as the delay in the identification of a specific cancer while 'disease management' referred to events such as the care of a terminally ill patient. Prescribing issues included prescribing an inappropriate dosage of medication. Almost as prevalent were issues precipitated by the patient or their relative (43, 22.5%) such as unnecessary medication requests and anger or upset at an aspect of their healthcare.

**Table 2:** Type and number of significant events identified (191 SEA reports)

**Significant Event Types**	**n**	**%**
Disease diagnosis and disease management *(e.g. missed or delayed diagnosis of cancer, terminal care pain management)*	46	24.1
Prescribing, dispensing and other drug issues *(e.g. wrong/inappropriate drug prescribed/administered, warfarin issue)*	46	24.1
Patient and relatives *(e.g. patient behaviour, anger or upset)*	43	22.5
Investigations and results *(e.g. incorrect results given to patient, results not acted upon)*	37	19.4
Communication *(e.g. lack of communication, unsuccessful communication)*	23	12.0
Administration *(e.g. complaint, breach of protocol)*	16	8.4
Medical records and confidentiality *(e.g. breach of confidentiality, wrong records accessed)*	15	7.9
Appointments and surgeries *(e.g. patient did not arrive issues, running late)*	12	6.3
Home visits and external care *(e.g. delay in arrival, visit request not done)*	10	5.2
Equipment *(e.g. computer search facility ineffective, difficulty accessing cupboard containing medical supplies)*	7	3.7
Miscellaneous *(e.g. difficulty in signing death certificate, change in partnership personnel)*	2	1.1
Health and safety *(e.g. staff injury, unsuccessful procedure for dealing with clinical waste)*	2	1.1

* More than one classification may have been accorded to a single SEA report.

### Reasons for occurrence

A classification summary with examples of the most frequently cited reasons by GPs as to why events occurred is outlined in Table 3. The most prevalent cause of events identified was that of individual health care professionals 'errors' relating to their knowledge and skills (62, 32.5%). Substandard communication issues between the practice and the patient or within and between care providers was the second most frequent cause of significant events occurring (58, 30.4%). Patient behaviour such as non adherence to medication or refusal to attend for investigations was also a significant contributory factor in over a quarter of reports (55, 28.9%).

**Table 3:** Reasons for occurrence of significant events and the number of reports identifying these occurrences (191 SEA reports)

**Reasons Given**	**n**	**%**
Individual health care professional 'errors' *(e.g. lack of knowledge of practice/hospital protocols, poor clinical task delivery)*	62	32.5
Communication *(e.g. substandard communication between practice and patient, substandard communication between practice and hospital/out of hours/other agencies)*	58	30.4
Patient and relatives *(e.g. negative patient behaviour, illness behaviour)*	55	28.9
Disease/diagnosis/management *(e.g. difficult diagnosis, incomplete history/examination)*	44	23.0
Administration *(e.g. poor task delivery, ineffective administrative system/protocol)*	32	16.8
Medication *(e.g. error writing/prescribing/administering (wrong drug dosage/formulation prescribed), no system/protocol to check for out of date emergency tray/bag medicines)*	23	12.0
Tests/investigations/results *(e.g. no sample tracking/record, delay in checking blood tests results)*	22	11.5
Patient records *(e.g. failure to check notes adequately, failure to record in notes)*	18	9.4
Equipment *(e.g. ineffective emergency buzzer system for staff to identify location of emergency, inadequate search facility on computer system)*	13	6.8
General practice protocols/systems/guidelines *(e.g. no formal protocol for checking BHCGs, no system for emergency bag tracking)*	8	4.2
Clinical behaviour *(e.g. doctor avoidance of addressing a difficult situation, lack of clinical leadership of patient review)*	8	4.2
Reasons for event undetermined	7	3.7
Appointments *(e.g. delay in being seen, not enough time with patients)*	6	3.1
Visits/external care *(e.g. change in out of hrs service, delay in attending house visit)*	3	1.6

• More than one classification may have been accorded to a single SEA report.

### External involvement in significant events

104 SEA reports (54.5%) described the direct or indirect involvement of other health and social care agencies in the significant event as follows: secondary care (58, 30.4%); community pharmacy (13, 6.8%); out-of-hours services (7, 3.7%) and social services (3, 1.6%); other [e.g. police or ambulance service] (32, 16.8%).

### Learning issues identified

182 reports (95.3%) identified learning needs, points or issues which required to be addressed as part of event analyses (Table 4). Over half of reports identified personal learning issues for the individual doctor who drafted the SEA report. These learning issues related mainly to 'generic' issues around diagnosis, clinical management and patient behaviour. Specific learning points relating to clinical knowledge in areas such as psychiatry or contraception were detailed in less than a sixth of reports.

**Table 4:** Type, range and number of learning issues identified from 191 SEA reports submitted

**Learning Issues identified**	**n**	**%**
Personal Awareness/Responsibilities and Change (*e.g. general issues on improving diagnosis and management, dealing with negative patient/family behaviour*)	98	*51.3*
Communication (*e.g. issues on communication with patient, issues on communication within team*)	54	*28.3*
Administration (*e.g. need for new/improved protocols/systems, staff training required*)	36	*18.9*
Clinical Knowledge (*e.g. psychiatry, contraception*)	30	*15.7*
Equipment/task aids/workspace (*e.g. become familiar with medical centre - whereabouts of drugs and equipment, the need to secure and check prescription pads*)	26	*13.6*
Case Notes (*e.g. the need for accurate detailed documentation, Read clinical notes*)	17	*8.9*
Whole Practice Awareness (*e.g. need for system to respond to emergency within practice, clarification of responsibilities*)	16	*8.4*
Medication/Prescription (*e.g. responsibilities for GP-secondary care interface prescribing, need for better supervision of PRHO prescribing*)	9	*4.7*
Patient/Carers (*e.g. effects of mental illness on carers, education required on self-management of asthma*)	6	*3.1*
Complaints (*e.g. dealing with complaints system, avoiding complaints being generated*)	4	*2.1*
GP and Partners Awareness (*e.g. the need for regular medication review with GPs, the importance of efficient and accurate results handling system*)	4	*2.1*
Health and Safety (*e.g. re-sheathing needles should not be undertaken, to ensure all clinical staff immunised against Hepatitis B*)	3	*1.6*

• 182 reports detailed at least one learning issue

• More than one learning point may have been reported in a single report.

### Actions agreed and implemented

154 reports (80.1%) demonstrated that change(s) had been agreed and implemented by at least one member of the primary care team as a result of the SEA (Table 5). 32 reports (16.6%) detailed a change(s) in which other GPs, nurses and health visitors in the GP practice (as well as the author of the report) were able to apply new or revised clinical knowledge and skills. In just under one sixth of reports this application of new knowledge, skills and changes in clinical behaviour was adopted by the reporting GP only.

**Table 5:** Type and range of actions taken from 191 SEA reports

**Changes implemented**	**n**	**%**
Clinical Team Disease Diagnosis and Management (*e.g*. raised clinical awareness/knowledge by dissemination to others then actioned, *raised procedural awareness and dissemination to others for action*)	32	16.6
Doctors Personal Skills/Behaviour/Knowledge application (*e.g. change in behaviour, application of knowledge*)	28	14.6
Communication (*e.g. improved communication with patients, improved communication between practice staff or between staff and doctors*)	26	13.6
Administration (*e.g. clarification of staff duties, member of staff designated to a particular role*)	26	13.6
Medication (*e.g. change to prescribing software, medication change highlighted on discharge script from hospital*)	24	12.6
Results/Investigations/Tests (*e.g. stop doing in-house tests*)	18	9.4
Patient Records (*e.g. improved recording in notes - paper or electronic, important patient information highlighted in notes - electronic or paper*)	16	8.4
Equipment and Workspace (*e.g. improved storage of equipment, face mask added to medical bag*)	15	7.8
Appointments (*e.g. increase appointment time for emergency surgery*)	3	1.6
Miscellaneous (*e.g. equipment & stocking within consulting room*)	2	1.0
Staffing/Premises Issue (*e.g. book used to document leave, revision of supervisory arrangements*)	2	1.0

• 154 SEA reports detailed actions to implement change

• More than one change may have been reported in a single report.

The methodology chosen to implement change was most often the development of new or adaption of existing protocols. This was detailed in 73 reports (44.5%)

### Levels of patient harm

48 SEA reports (25.1%) described incidents which led to patient harm (Table 6). A further 109 reports (57.1%) outlined circumstances which had the potential to cause patient harm but were either prevented or ran to completion without harm occurring ('near misses'). A minority of reports (34, 17.8%) did not involve incidents which could have compromised patient safety.

**Table 6:** Level of patient harm as determined by NPSA grading system [27]

**NPSA Severity Grading**	**GP Principals** **(n)**	**GP Registrars** **(n)**	**Total** **n (%)**
**No Harm - Impact Prevented** *Any patient safety incident that had the potential to cause harm but was prevented, resulting in no harm to people receiving NHS-funded care*	24	28	52 (27.2)
**No Harm - Impact Not Prevented** *Any patient safety incident that ran to completion but no harm occurred to people receiving NHS-funded care*	33	24	57 (29.8)
**Harm - Low** *Any patient safety incident that required extra observation or minor treatment and caused minimal harm, to one or more persons receiving NHS-funded care*	7	7	14 (7.3)
**Harm - Moderate** *Any patient safety incident that resulted in a moderate increase in treatment and which caused significant but not permanent harm, to one or more persons receiving NHS-funded care*	9	13	22 (11.5)
**Harm - Severe** *Any patient safety incident that appears to have resulted in permanent harm to one or more persons receiving NHS-funded care*	6	3	9 (4.7)
**Harm - Death** *Any patient safety incident that directly resulted in the death of one or more persons receiving NHS-funded care*	2	1	3 (1.6)
**Not applicable** *Non-patient safety incidents*	18	16	34 (17.8)

**TOTAL**	99	92	**191**

Of the 48 reports of patient harm, 42 (87.5%) described the direct or indirect involvement of health care teams or agencies external to the general practice as well as members of the practice team. In comparison, of the 109 reports that had the potential to result in patient harm but did not, a total of 62 involved health care teams or agencies (56.9%) external to the practice [difference 30.6%; 95% CI 17.4 to 43.8; P < 0.001].

The involvement of staff in the SEAs where learning issues were identified and where change was implemented are shown in Table 7. Practice managers, nurses and administration staff were significantly less likely to be involved in both the learning and the implementation of change from the analysis of a significant event which resulted in patient harm compared with events which did not cause harm.

**Table 7:** Involvement of healthcare professionals in the learning and implementation of change from undertaking a SEA.

**Learning Needs Identified** (182/191 = 95.3%)	Reporting GP	Partners	Practice Manager	Practice Nurse	Administration. Staff	Health Visitor	District Nurse	Comm. Pharmacist	Hospital
Patient Harm **(n = 44)**	44 (100%)	13 (29.6%)	2 (4.6%)	2 (4.6%)	4 (9.1%)	0 (0%)	0 (0%)	1 (2.3%)	0 (0%)

Non-Patient Harm **(n = 138)**	138 (100%)	57 (41.3%)	35 (25.4%)	29 (21.0%)	29 (21.0%)	2 (1.4%)	0 (0%)	6 (4.3%)	1 (0.7%)

*Difference (95% Confidence Intervals) P-value*	*NA*	*11.8 (4-27)* *P = 0.14*	*21 (11-30)* *P < 0.01*	*16 (7-26)* *P < 0.01*	*12 (1-23)* *P = 0.03*	*NA*	*NA*	*NA*	*NA*

**Action Taken** (154/191 = 80.6%)	Reporting GP	Partners	Practice Nurse	Admin. Staff	Health Visitor	District Nurse	Comm. Pharmacist	Hospital	

Patient Harm **(n = 34)**	34 (100%)	28 (82.4%)	7 (20.6%)	3 (8.8%)	2 (5.9%)	0 (0%)	0 (0%)	0 (0%)	1 (2.9%)

Non-Patient Harm **(n = 120)**	117 (97.5%)	87 (72.5%)	47 (39.2%)	33 (27.5%)	47 (39.2%)	2 (1.7%)	4 (3.3%)	0 (0%)	2 (1.7%)

*Difference (95% Confidence Intervals) P-value*	*NA*	*10 (5-25)* *P = 0.20*	*18(2-35)* *P = 0.02*	*19(6-31)* *P = 0.003*	*33(21-45)* *P < 0.01*	*NA*	*NA*	*NA*	*NA*

• Because of small numbers no direct comparison was made between heath visitors, district nurses, community pharmacists and hospitals.

## Discussion

### Main findings

The types of significant events described in this study are consistent with the broad range of clinical and administrative events previously identified in similar general practice studies [24,34]. The majority of events had the potential to cause patient harm, while one quarter described incidences of patient harm. Several of the major underlying reasons why significant events occurred, such as knowledge and skills errors or communication difficulties are consistent with previously reported reasons why errors and adverse events occur in both UK and international family practice [7,8,11,12]. Although learning issues are identified in the majority of SEA reports these frequently relate to non-specific personal learning issues which do not appear to be shared with the practice team. There also appears to be limited direct involvement of the practice team members in implementing the changes required from the SEA. If, however, a significant event has resulted in patient harm then medical colleagues tend to be involved in the implementation of change in the great majority of cases.

### Study limitations and strengths

The reports voluntarily submitted to the SEA peer feedback model described may not be representative of those undertaken by the GP population as they are likely to be highly selective [31]. The reports reviewed were judged by trained peers to be 'satisfactory' event analyses, while a substantial minority was considered 'unsatisfactory'. This provides an element of verification and assurance of SEA quality, which does not exist in other similar studies. However, this limits the generalisation of the findings since no comparison was made between the types of events and the subsequent learning issues and change implemented between those reports deemed 'satisfactory' and 'unsatisfactory'. There could also be selection bias as SEA judged 'unsatisfactory' may describe important quality and safety issues but be difficult for GPs to describe within the report format. SEA reports are written retrospectively normally by a single author who in most cases acts as a proxy for the practice. Personal recollections may therefore be affected by recall bias or misinterpretation of reasons for event occurrence or decisions made. The study may have been strengthened by undertaking a more in-depth textual analysis of a sample of SEA reports. This could have enabled the authors to identify (and subsequently amend) elements of the reporting format that inhibits analysis of particular events and limits reflective team based learning.

### Context and Implications

#### Types of Events

SEA was originally proposed as an adjunct to the traditional quantitative approach to audit that allowed GP practices to investigate specific areas of care not accessible by this method [24]. The range of significant events identified for analysis in this study confirms the potential for SEA to examine a breadth of patient-safety related subject matter which appears largely unlikely to be captured by criterion or cohort based audit. Although there is much to be learned from 'good practice' it is apparent that GP teams choose in the vast majority of cases to examine events that could highlight sub-optimal care presumably because they find these events are a more valuable learning experience [31]. The most common clinical areas for analysis were cancers and acute psychiatric events and this most probably reflects their role as 'marker' events identified by the General Medical Services contract (GMS) in the UK as being worthy of SEA [29]. The benefits of using these 'marker' events is that they can provide opportunities for prevention, early detection and inform on the process of care [35]. They may also highlight previously unknown learning needs. However there may be an opportunity-cost to analysing these 'prescriptive' events over self-selected events of potentially greater importance. The frequent choice of events that relate to disease diagnosis and management and those that involve prescribing and drug issues are consistent with GPs selecting topics that reflect the routine case work of general practice both in the UK and in other similar international healthcare systems [12]. It also reflects the subject choice of SEA in other parts of the UK [34].

#### Reasons for occurrence

This study found that the two most common reasons cited for the significant event having taken place were self-reported individual errors by the doctor and communication issues. The role of individual error may reflect appropriate insight on the part of the doctor. It is more likely, however, that it could indicate a lack of understanding of the deeper systems-based factors which contribute to these errors or violations since it is known that flawed health care systems rather than just the specific actions of individuals are often the underlying causes of patient safety incidents [36]. Such lack of understanding could be addressed through local initiatives such as existing community health partnerships (CHPs) which provide educational sessions for all relevant primary healthcare staff or individual learning through continuing professional development.

Given that these SEA reports are submitted as part of an educational exercise for the GP, it may be that the doctors feel they will learn more by analysing significant events attributable to themselves than events more directly attributable to others or team members. Additionally GPs are known to submit events that they feel responsible for as a form of 'personal catharsis '[31].

Patient behaviour was thought to be an underlying factor in over a quarter of significant events, which has rarely been cited as a key reason for significant event occurrence in general practice [37]. It does not necessarily mean that there is 'blame' attached to the patients' role. Significant events can be influenced by a host of factors including illness behaviour or lack of knowledge on the part of the patient or their carers' or poor lines of communication between the practice and the patient. Buetow and Elwyn suggest that events, which may in part be attributable to patients, need to be understood in the context of their individual and social circumstances [38]. For instance, a patient may contribute to a significant event because an agreed action at a consultation cannot then be carried through by the patient - such as taking time off employment to attend out patient clinics. Patients may also make an informed choice that leads to a significant event, and in such circumstances may be 'morally' responsible for errors that they make [39]. However, the GP or the practice may contribute if they do not agree preferred management options with the patient. In such circumstances, patients may feel that they lack 'enablement' or 'partnership' in the decision making process within the consultation. This can highlight training issues for the GP in their consultation techniques.

#### External Involvement in SEA

A study in one Primary Care Trust in England found that nearly 19% of significant events originated beyond the general practice unit [34]. Although our study did not look at place of origin, the finding that over half of events involved an agency external to the practice highlights the potential for multi-disciplinary and multi-agency learning and collaboration in event analyses. This finding may also demonstrate that many GP teams are prepared to investigate what could be interpreted as difficult-to-resolve "interface" issues. Alternatively there could be a degree of 'blame-shifting' attached to the event analyses.

#### Learning and change

The role of SEA as a reflective learning technique has been highlighted [24,25]. The findings provide further evidence of this but it is of note that much of the learning would appear to be personal to the SEA report author. There may be a reluctance to share this with colleagues if the GP was professionally embarrassed or felt that the learning point was not of sufficient interest to others. It is also possible that there are difficulties with team dynamics or interpersonal relationships within the GP team. This would limit the potential of SEA but could be addressed through training in team based learning for all relevant members of the primary care unit. Another interpretation is that the authors failed to document that they shared their learning with other members of the practice team.

Although reflection and learning is recognised as an important part of improving the quality of healthcare [40] it is the application of this learning into sustained change that will enhance the quality of each individual's care. It is unclear if the learning and change described in these SEA reports led to sustained improvement in health care practices. Of additional concern is the substantial minority of reports not included in the study as they were considered unsatisfactory by trained peers. The opportunities for professional learning from events that may involve doctors' mistakes or other healthcare errors may be lost due to a failure to fully understand and apply the SEA technique [31,41]. This may imply that SEA training - which is mainly (though not exclusively) undertaken as part of postgraduate medical education - needs to be encouraged through both teaching of GP registrars and through GP appraisal [28]. The evidence that SEA can improve the quality of care, and enhance patient safety in particular, is limited [25]. However, perhaps it should be seen in the context of a raft of other quality improvement approaches - such as audit, prescribing reviews, referral analysis, complaints review and the nGMS contract itself - that are available to GPs and their teams as part of a multi-method approach to reducing harm and minimising risk.

#### Patient safety

The small percentage of SEA reports that involved severe harm or death is consistent with other recent UK data on the severity of events analysed by GPs [31]. The NPSA recommends that a full Root Cause Analysis (RCA) should be conducted in these cases rather than SEA [27]. RCA is a more intensive and structured investigation process which is normally led by a group of trained health care professionals who are independent and external to the event [42]. However, a raft of barriers potentially militates against the use of RCA in general practice: the independent contractor status of GPs may mean they are not obliged to co-operate with external RCA investigators; the time and cost implications of training GP team members in RCA would be a major obstacle, while the use of face-to-face interviews as a data collection method when applying the technique could be potentially divisive in small, close-knit teams. Although it is clear that severe events occur in general practice, there does not appear to be evidence that RCA is routinely applied. Policymakers may need to reconsider the use of RCA in these situations. In addition, SEA is only one method to identify and investigate patient safety issues. Criterion audit, the use of trigger tools, patient surveys and interviews, case note review, and 'sentinel event' monitoring all have a role in identifying adverse events and errors [35,43,44]. These methods should be considered complementary and healthcare teams need to be able to apply these techniques independently since there is often no overlap in their identification of different patient safety issues [44]. There may be practical issues for teams in allocating time and resources to both apply and integrate these methods. However, it is important that the interface between SEA and other patient safety and healthcare quality improvement techniques is established. This will help ensure that SEA is undertaken appropriately.

With one in four events involving some form of harm, (nearly all of which involve external agencies) it is also apparent that GPs are prepared to confront potentially difficult issues that may not reflect well on them or their practices. It is acknowledged that professional 'shame' can inhibit the reporting of relevant safety issues [45]. However, efforts to minimise the emotional consequences of such feelings can be achieved through recognition of the underlying contributing factors to these incidents [46]. Sharing such events to improve the quality of care rather than punish individuals can act as a driver to quality improvement [47]. The majority of events had the potential to cause patient harm but did not actually do so. GPs have been encouraged to identify and use these types of 'near misses' to highlight learning and safety issues in practice rather than wait till a patient inevitably suffers harm.[29,35] Given the range of outcomes outlined in the reports, if properly applied, the SEA technique would appear to be well placed to inform and educate on a wide range of patient safety-related issues in general practice.

Although SEA is encouraged as a team-based activity [24,25] if the event in question involved patient harm then non-medical staff were less likely to be involved in the learning and change as a result of the analysis. As most GPs will involve staff only where they deem it appropriate [48] this is likely to represent a pragmatic decision on the part of GPs to form a smaller, more relevant group when clinical care issues are involved. Alternatively there may be a reluctance to open up discussion to the wider primary care team if the doctor perceives patient harm to have been in some way related to their own professional practice.

Significant events are often described as a rich resource that could aid both local and national reporting and learning systems[27]. Indeed most of the patient safety incidents amongst the SEA reports in this study could be relatively easily applied to the NPSA reporting template and other international taxonomies [13,49]. The narrative aspect of SEA should serve to add depth to basic factual details applied to reporting systems in health care and thus could potentially offer much more information than most incident reporting forms [43,47]. In addition, the patient harm described in this study may be an under-representation of the true volume of patient harm identified by SEA since GPs are selective in the type of significant events that they choose to submit to this peer review model and those GPs who do submit their SEA for feedback may not be representative of their GP colleagues. Given the large scale non-engagement of GPs in incident reporting systems [30] it may be appropriate to encourage the notification and sharing of these reports to inform the patient safety agenda at the local and national level.

## Conclusion

The study adds to the limited evidence of the potential of SEA to improve healthcare quality and safety. It is applied to investigate and learn from a wide variety of quality-related issues identified by GP teams, including those resulting in patient harm and which often involve other health care agencies. Learning and change reportedly occur but more research is required to establish if sustainable improvement is possible. Innovative methods of disseminating learning and change to the wider primary care environment are also required, while exploration of the linkage between SEA and improving the engagement of GP teams in local or national patient safety reporting systems should also be elucidated.

## Competing interests

The authors declare that they have no competing interests.

## Authors' contributions

JM contributed to the conception and design of the study, the collection, analysis and interpretation of data. NB contributed to the analysis and interpretation of data. ML contributed to the conception of the study and critical discussion of the analysis. PB contributed to the conception and design of the study and the analysis and interpretation of data. All authors contributed to the drafting and write up of the manuscript.

## Funding

The study was funded by NHS Education for Scotland.

## Pre-publication history

The pre-publication history for this paper can be accessed here:


